# Supplementary material for: Phenotypic plasticity in cell elongation among closely related bacterial species
Source: Nat Commun. 2025 Jun 2;16:5099. doi: 10.1038/s41467-025-60005-y (PMC12130487; doi:10.1038/s41467-025-60005-y)
Supplement: Supplementary file 1 — Supplementary Information [file 41467_2025_60005_MOESM1_ESM.pdf]

Supplementary Information

Phenotypic plasticity in cell elongation among closely related bacterial species

Marie Delaby[1,#], Liu Yang[1,2,#], Maxime Jacq[1], Kelley A Gallagher[1,3], David T Kysela[1], Velocity Hughes[1,4], Francisco Pulido[5], Frederic J. Veyrier[5], Michael S. VanNieuwenhze[6], Yves V. Brun[1,7*]

# contributed equally

*Address correspondence to Yves V. Brun, yves.brun@umontreal.ca

Supplementary Fig. 1 to 8

Supplementary Note 1

Supplementary Tables S1 to S4

Supplementary References

Supplementary Fig. 1

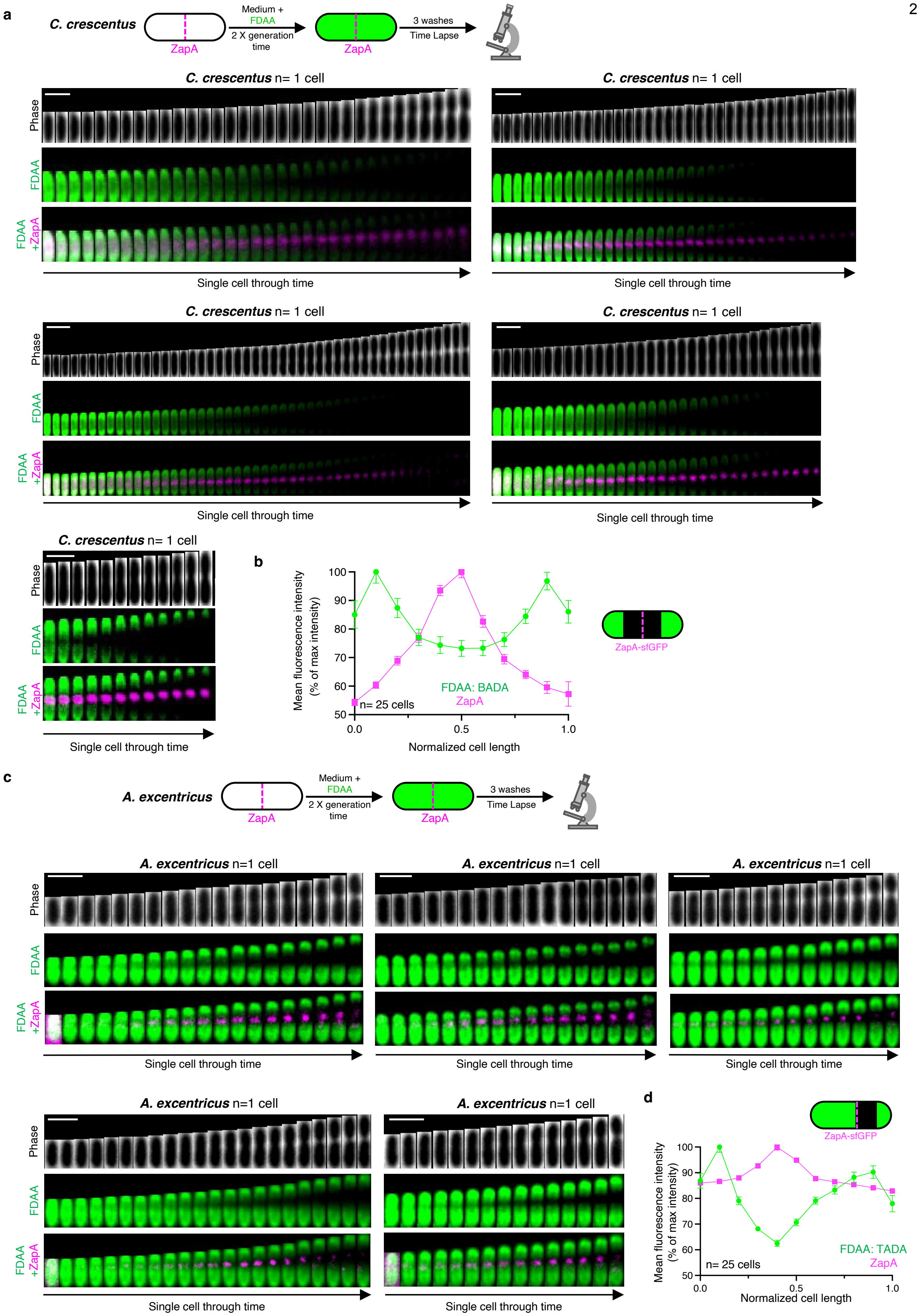

Supplementary Fig. 1. *C. crescentus* and *A. excentricus* have different patterns of PG synthesis

(a) Schematic of the pulse-chase experiment using the FDAA BADA (green) in *C. crescentus* cells expressing ZapA-mCherry (magenta). Kymographs from five different cells from this pulse-chase experiment are shown. Scale bar 2 µm (vertical, cell length). **(b)** Graphs showing the fluorescence intensity of the FDAA and the ZapA-mCherry (magenta) against their relative positions along the cell length for 25 cells. **(c)** Schematic of the pulse-chase experiment using the FDAA TADA (green) in *A. excentricus* cells expressing ZapA-sfGFP (magenta). Kymographs from five different cells from this pulse-chase experiment are shown. Scale bar 2 µm (vertical, cell length). **(d)** Graphs showing the fluorescence intensity of the FDAA and the ZapA-sfGFP (magenta) against their relative positions along the cell length for 25 cells. Source data are provided as a Source Data file.

Supplementary Fig. 2

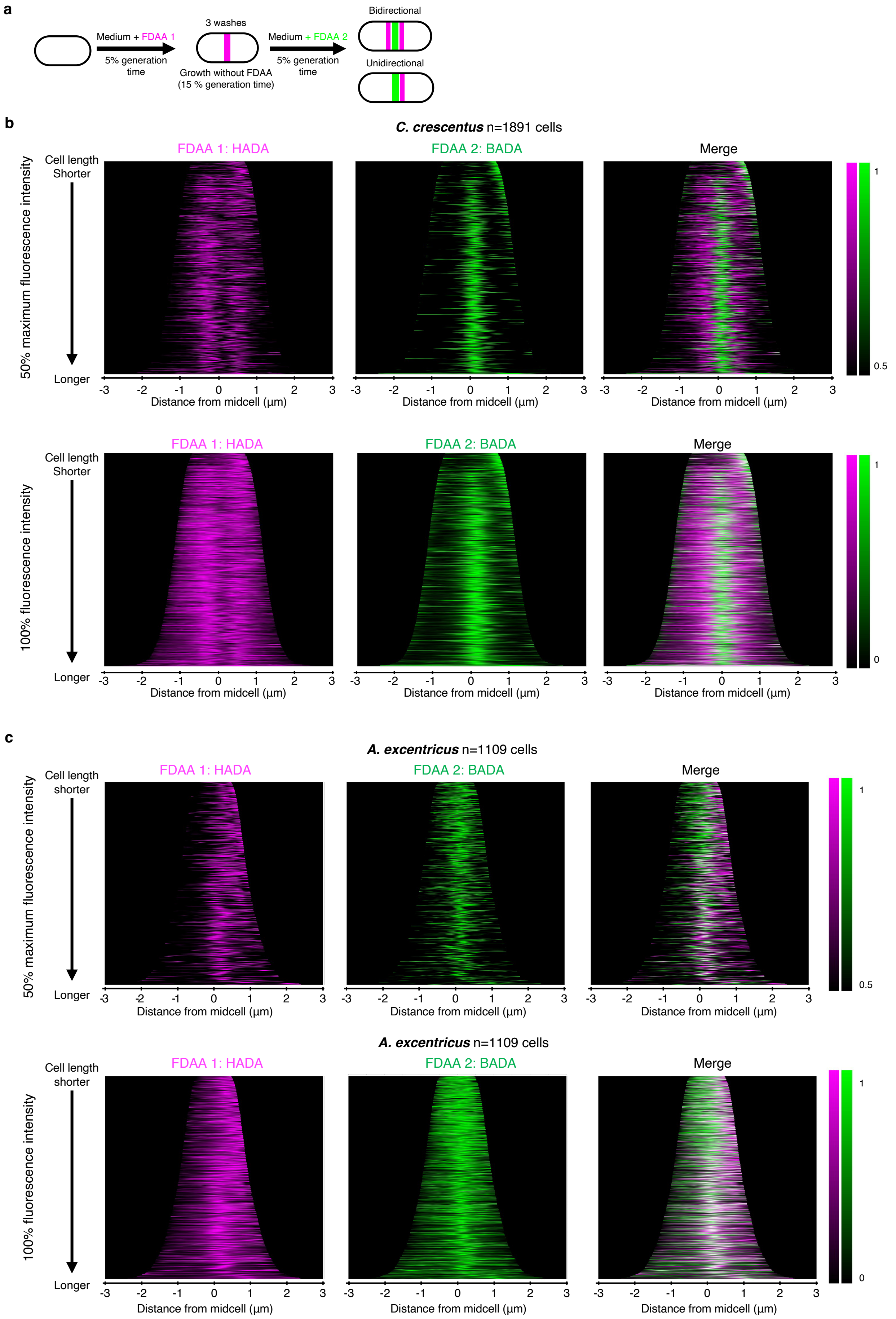

Supplementary Fig 2. Demographs from dual short-pulse labeling with FDAAs in *C. crescentus* and *A. excentricus*.

(a) Schematic depicting the dual short-pulse experiment. Cells were first labeled with one FDAA for 5% of their generation time (HADA for *C. crescentus* and TADA for *A. excentricus*), washed with PYE to remove free FDAA, allowed to grow for 15% of their generation time and then labeled with a second FDAA (BADA) for 5% of their generation time, washed again, and imaged with phase and fluorescence microscopy. **(b-c)** Demographs showing the fluorescence intensities of both FDAA signals in **(b)** *C. crescentus* cells and **(c)** *A. excentricus* cells. Demographs are presented for each FDAA independently, as well as together on the same graph. Cells were arranged by length with the maximum fluorescence intensity of the FDAA signal to the left. 50% of the maximum fluorescence intensities (*top*) and 100% of the fluorescence intensities (*bottom*) are shown. The first FDAA is represented in magenta and the second FDAA in green.

Supplementary Fig. 3

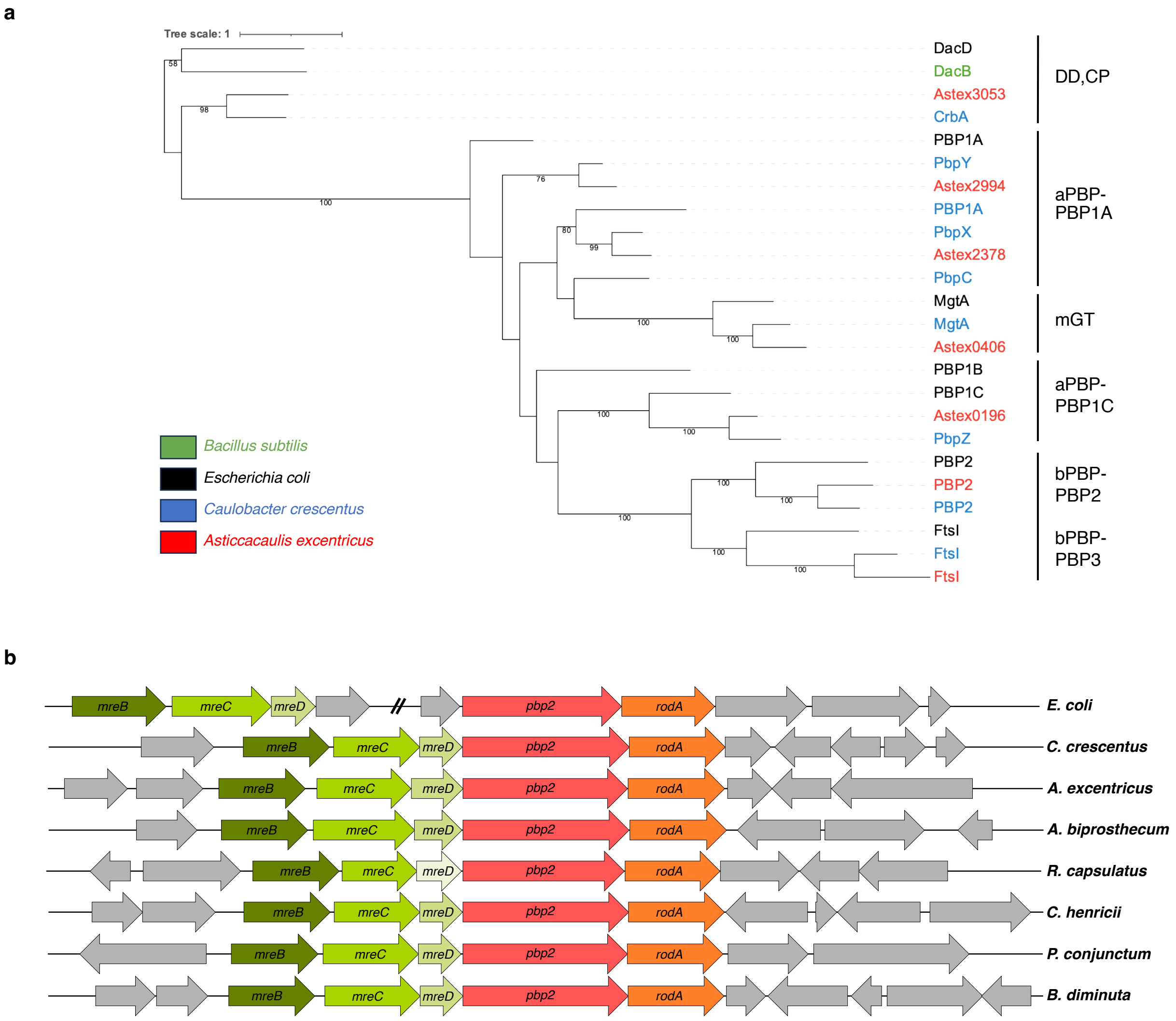

Supplementary Fig. 3. Sequence alignment of PBPs from different species and genomic organization of the *pbp2* and *mreB* genes.

(a) Phylogenetic tree of PBP sequences from *E. coli*, *B. subtilis*, *C. crescentus* and *A. excentricus*. Each species is represented by a specific color: black for *E. coli*, blue for *C. crescentus*, red for *A. excentricus*, and green for *B. subtilis*. aPBP: class A penicillin-binding protein; bPBP: class B penicillin-binding protein; D,D-CP: D,D-carboxypeptidase; and mGT: mono-glycosyltransferase. Bootstrap values >50 % are indicated at their respective nodes (based on 100 replicates). The final tree was formatted using iTol[1]. **(b)** Overview of the genomic organization of *pbp2* and *mreB* genes in selected species. The figure displays the genomic arrangement of the *pbp2* and *mreB* loci across the species of interest, presented from top to bottom: *E. coli*, *C. crescentus*, *A. excentricus*, *A. biprosthecum*, *R. capsulatus*, *C. henricii*, *P. conjunctum*, and *B. diminuta*. The genes are color-coded according to their type: *pbp2* is represented in red, and *rodA* in orange. *mreB*, *mreC*, and *mreD* genes are shown in shades of green, with *mreD* from *R. capsulatus* depicted in a lighter green to indicate that it did not meet the identity and e-value cut-off. All other neighboring genes are shown in gray to indicate non-relevant or less conserved regions. The length of each arrow and the spacing between them reflect the gene sizes and intergenic distances in nucleotides.

Supplementary Fig. 4

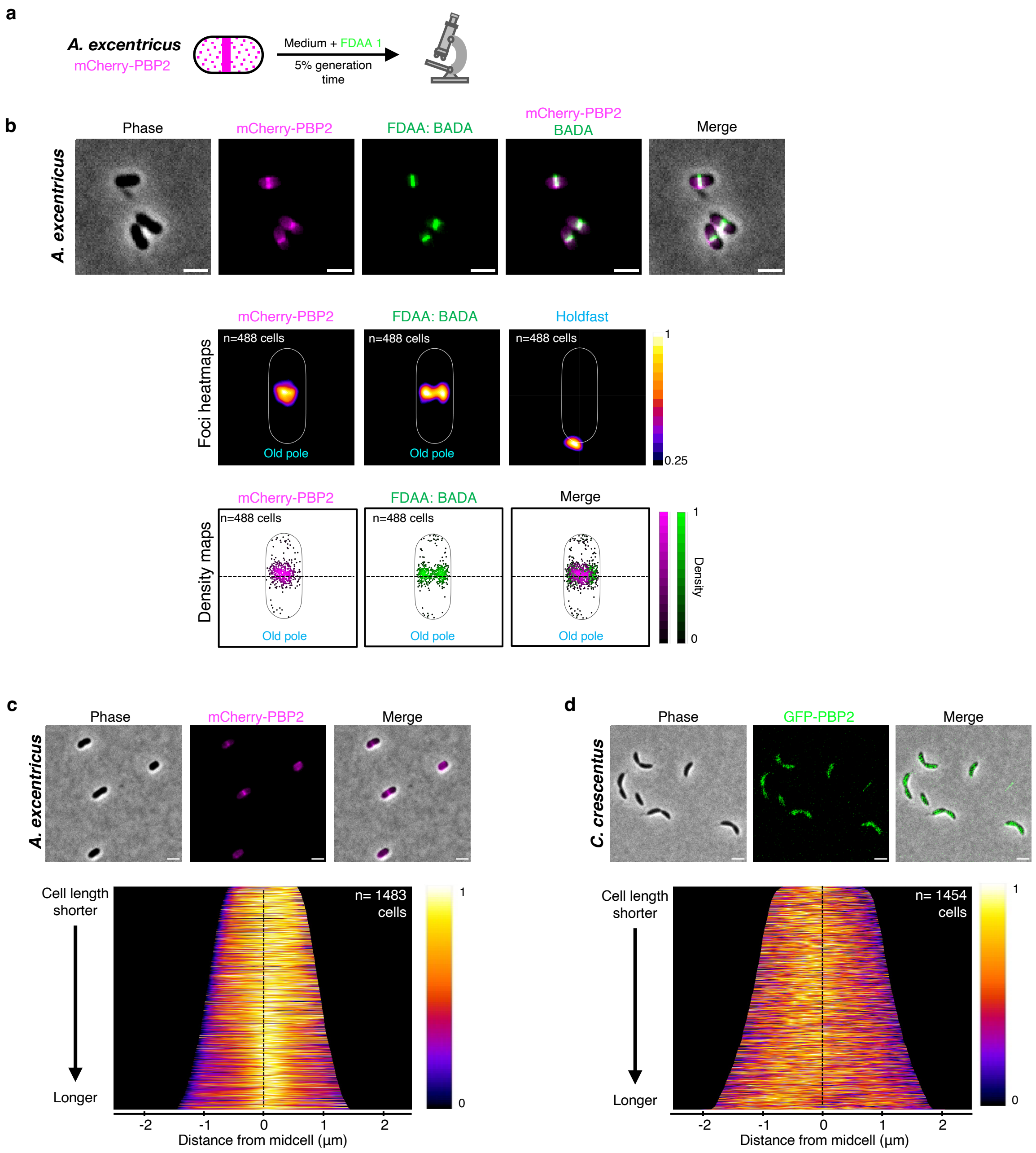

Supplementary Fig. 4. PBP2 localizes close to the midcell in *A. excentricus* while PBP2 is dispersed in *C. crescentus.*

(a) Short-pulse FDAA (BADA) labeling of *A. excentricus* cells expressing mCherry-PBP2. A schematic depicting the short-pulse experiment is shown. Cells were labeled with 500 µM BADA for 5% of their generation time, fixed with 70% ethanol and imaged. **(b)** *Top*: Representative images of the short-pulse labeled *A. excentricus* mCherry-PBP2 cells are shown (n = 3 biological replicates). *Left to right*: Phase channel, mCherry-PBP2, BADA, and merged images with WGA labeling. Scale bar: 2 µm. *Middle*: Heatmaps of mCherry-PBP2 and BADA foci at the population level are shown. *Bottom*: Density maps of mCherry-PBP2 and BADA foci at the population level are shown, with the black line indicating the midcell. **(c)** Subcellular localization of mCherry-PBP2 in *A. excentricus*. *Top*: Representative images are shown with phase, mCherry-PBP2, and merged images (n = 3 biological replicates). Scale bar: 2 µm. *Bottom*: A demograph showing the localization of the fluorescence intensity of mCherry-PBP2 at the population level, with each cell oriented such that the pole with the maximum fluorescence intensity is to the left. **(d)** Subcellular localization of GFP-PBP2 in *C. crescentus*. *Top*: Representative images are shown with phase, GFP-PBP2, and merged images (n = 3 biological replicates). Scale bar: 2 µm. *Bottom*: A demograph showing the localization of the fluorescence intensity of GFP-PBP2 at the population level, with each cell oriented such that the pole with the maximum fluorescence intensity is to the left.

Supplementary Fig. 5

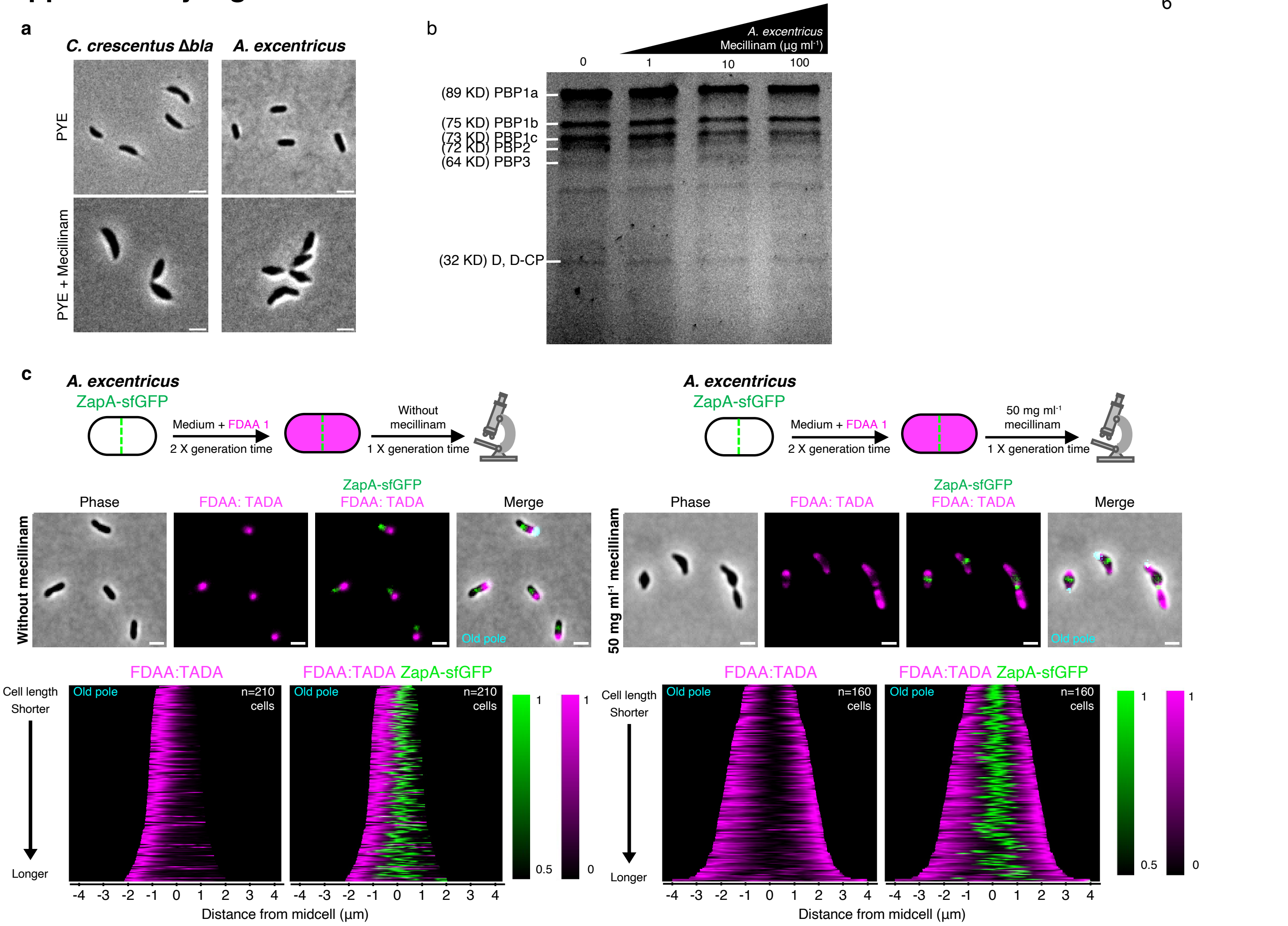

Supplementary Fig. 5. Mecillinam targets PBP2 in *C. crescentus* and *A. excentricus*

(a) Phase contrast images of the Δ*bla* β-lactam-sensitive strain of *C. crescentus*, and *A. excentricus* WT cells treated with or without mecillinam (50 μg ml^{-1}). **(b)** SDS-PAGE gel image for mecillinam titration against the different PBPs in *A. excentricus*. Whole cells were treated with various concentrations of mecillinam and subsequently labeled with Boc-FL prior to SDS-PAGE. **(c)** Demograph analysis of pulse-chase experiments in *A. excentricus* ZapA-sfGFP cells with (*right*) or without (*left*) mecillinam treatment. Whole-cell PG was labeled with TADA (magenta) over two generations, followed by washing and growth with or without mecillinam (50 μg ml^{-1}) over one generation before imaging. Representative phase, fluorescence (TADA and TADA overlaid with ZapA-sfGFP) and merged images with WGA fluorescence from both conditions are shown. Scale bar: 2 μm. Demograph showing *A. excentricus* ZapA-sfGFP cells with (*right*) or without (*left*) mecillinam treatment. Loss of FDAA labeling shows sites of PG synthesis/remodeling. Cells are arranged by length, with each cell oriented so that the old pole is set to the left.

Supplementary Fig. 6

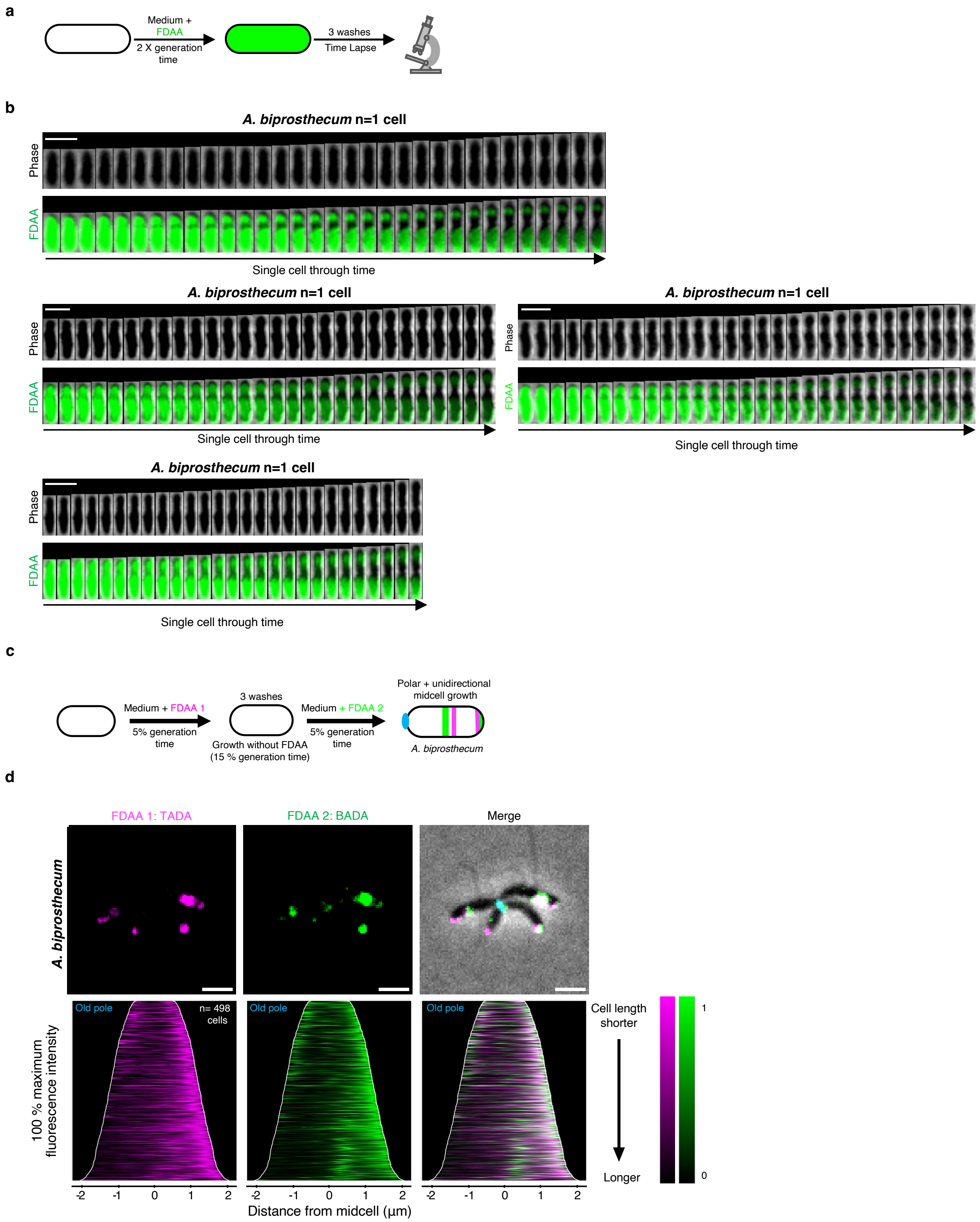

Supplementary Fig. 6. Pulse-chase and dual short-pulse FDAA labeling experiments reveal that *A. biprosthecum* shows both polar and unidirectional midcell growth.

(a) Schematic of the pulse-chase experiment. Whole-cell PG was labeled with 500 µM FDAA (TADA, green) over two generations, followed by washes with PYE to remove free FDAA from the medium. Subsequent growth in the absence of FDAA was followed by time-lapse microscopy. During this chase period, the loss of FDAA signal corresponds to new PG synthesis/turnover. **(b)** Representative cells from the pulse-chase experiment (n = 3 biological replicates). Five different *A. biprosthecum* cells and corresponding kymographs are shown. Scale bar: 2 µm (vertical, cell length). **(c)** Schematic depicting the dual short-pulse experiment. Cells were first labeled with one FDAA (TADA, magenta) for 5% of their generation time, washed with PYE to remove free FDAA, allowed to grow for 15 % of their generation time, and then labeled with a second FDAA (BADA, green) for 5% of their generation time, washed again, and imaged with phase and fluorescence microscopy. **(d)** *Top*: Representative images (n = 3 biological replicates) showing each FDAA individually or merged with phase and old pole labeling with WGA (cyan). Scale bar: 2 µm. *Bottom*: Demographs of the FDAAs showing the full range of their fluorescence signals, individually or merged in the same graph. Cells were arranged by length with the old pole (labelled with WGA) to the right.

Supplementary Fig. 7

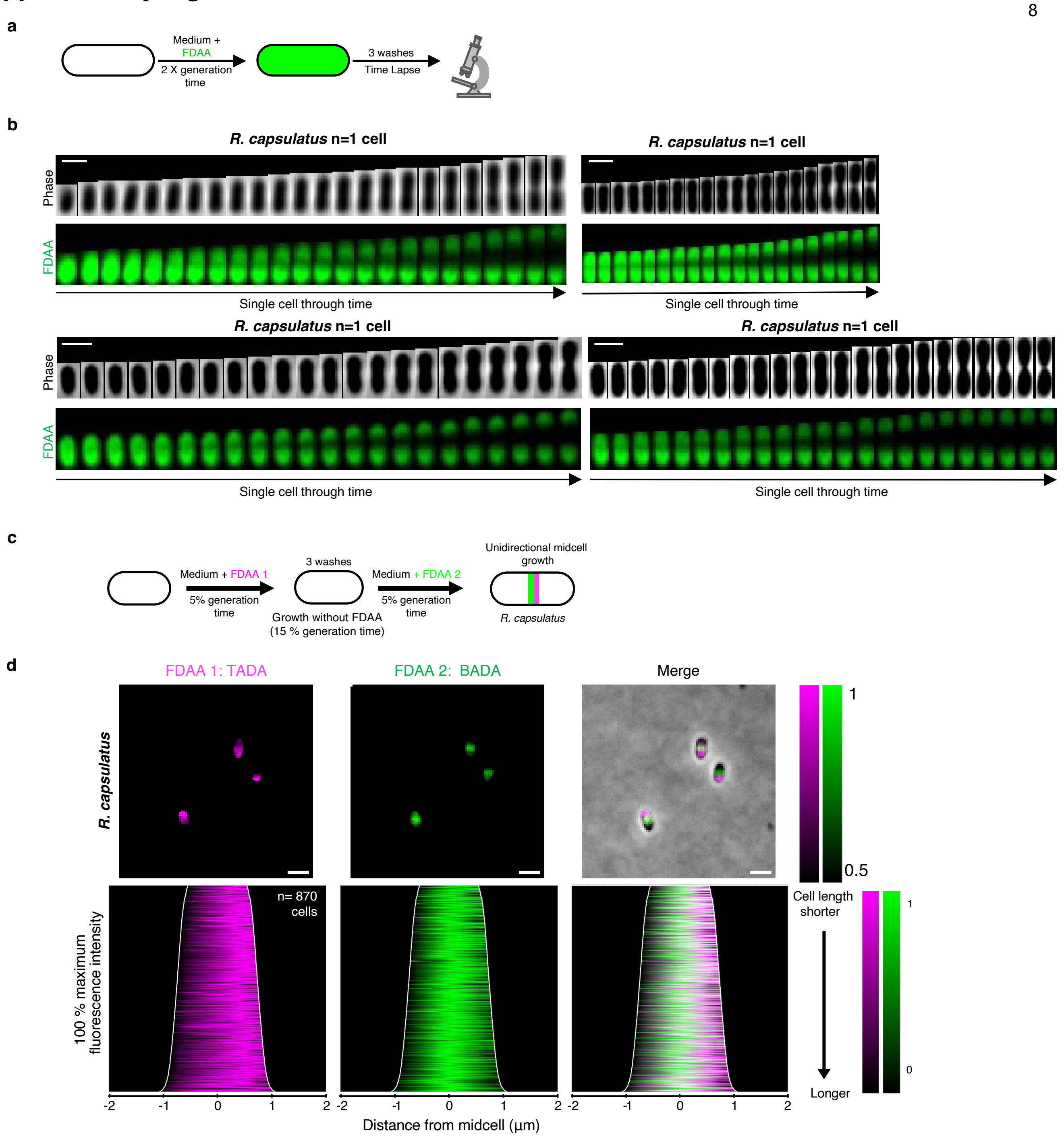

Supplementary Fig. 7. Pulse-chase and dual short-pulse FDAA labeling experiments reveal that *R. capsulatus* shows unidirectional midcell growth.

(a) Schematic of the long-pulse and chase experiment. Whole-cell PG was labeled with 250 µM FDAA (TADA, green) over two generations, followed by washes with PYS to remove free FDAA from the medium. Subsequent growth in the absence of FDAA was followed by time-lapse microscopy. During the chase period, the loss of FDAA signal corresponds to new PG synthesis/ turnover. **(b)** Representative cells during from the pulse-chase experiment (n = 3 biological replicates). Four different *R. capsulatus* cells and corresponding kymographs are shown. Scale bar: 2 µm (vertical, cell length). **(c)** Schematic depicting the dual short-pulse experiment. Cells were first labeled with one FDAA (TADA, magenta) for 5% of their generation time, washed with PYS to remove free FDAA, allowed to grow for 15% of their generation time and then labeled with a second colored FDAA (BADA, green) for 5% of their generation time, washed again, and imaged with phase and fluorescence microscopy. **(d)** *Top*: Representative images showing each FDAA individually or merged with phase and old pole labeling with WGA (cyan) (n = 3 biological replicates). Scale bar: 2 µm. *Bottom*: Demographs of the FDAAs showing the full range of their fluorescence signals, individually or merged in the same graph. Cells were arranged by length with the maximum fluorescence intensity to the left.

Supplementary Fig. 8

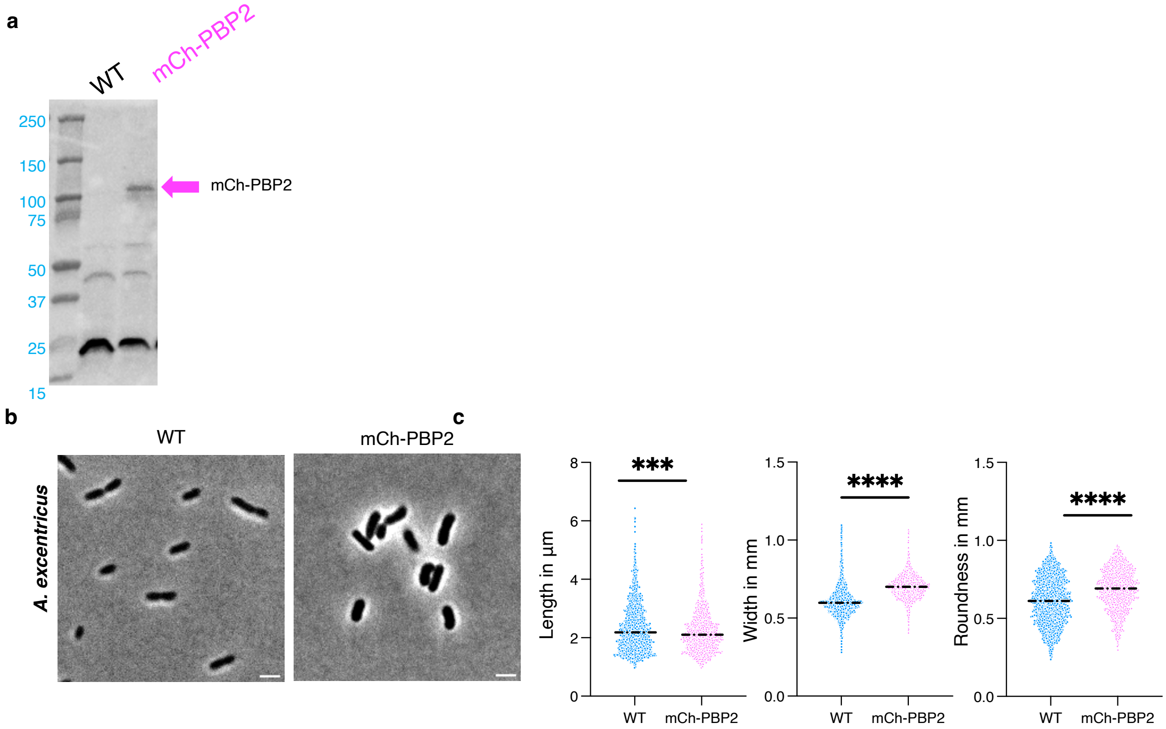

d

Strain	Mutation
mCh-PBP2	FtsW : L34V and L35V

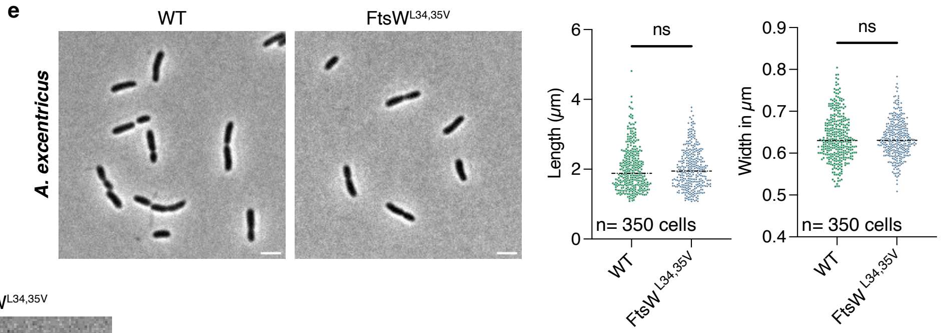

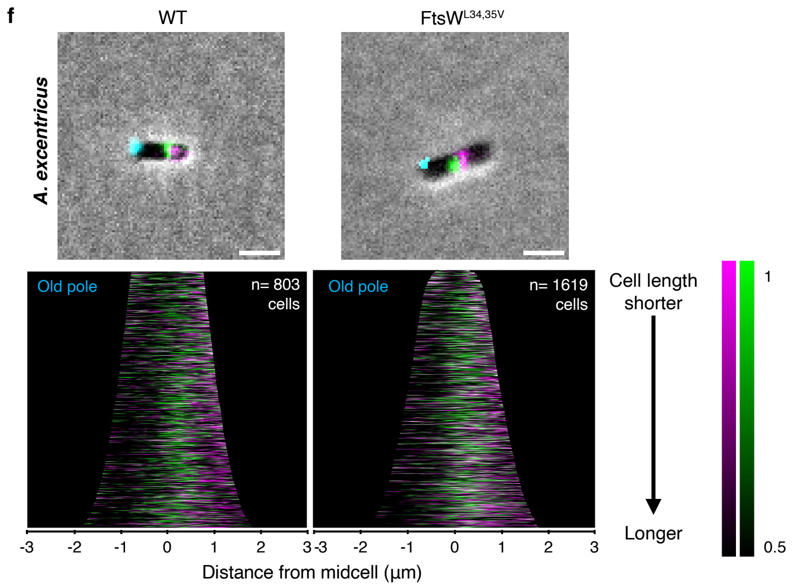

Supplementary Fig. 8. Validation of the mCherry PBP2 fusion in *A. excentricus*.

(a) Western blot analysis of WT and mCh-PBP2 cells. **(b)** Representative images of WT and mCh-PBP2 cells (n = 3 biological replicates). Scale bars: 2 µm. **(c)** Dot plots comparing the length, width, and roundness of cells expressing the mCherry-PBP2 fusion, compared to WT. Statistical analysis was performed using unpaired two-tailed t-tests with Welch's correction. Significant differences were observed for all parameters: ***P = 0.0002 (length), ***P < 0.0001 (width and circularity), relative to WT. **(d)** Results of whole-genome sequencing for strain *mCherry-pbp2*. The *mCherry-pbp2* strain carries two missense mutations (L34V and L35V) in the *ftsW* gene. **(e)** Phenotype of the *ftsW* L34V and L35V mutations backcrossed into WT. Representative phase-contrast images and dot plots comparing the length and width of *ftsW*$^{L34V\ L35V}$ to WT (n=350 cells). Scale: 2 µm. No significant differences were observed for either length (P = 0.8269) or width (P = 0.4604) based on unpaired two-tailed t-tests with Welch's correction.**(f)** *Top*: Representative images are shown (n = 3 biological replicates). *Left*: merge image of FDAA 1 TADA, FDAA 2 BADA, and phase contrast of WT cells. *Right*: merge image of FDAA 1 TADA, FDAA 2 BADA, and phase contrast of *ftsW*^{L34VL35} cells. Scale bars: 2 µm. *Bottom*: Demographs showing the fluorescence intensity of both FDAA signals in WT and ftsW L34V L35V cells. Cells were arranged by length with the old pole (labelled with WGA) to the right. 50% of the maximum fluorescence intensities are shown. Source data are provided as a Source Data file.

Supplementary Note 1:

To assess the functionality of our mCherry-PBP2 fusion in *A. excentricus*, we first confirmed the expression of the full-length fusion protein by Western blot analysis, which detected a band corresponding to the expected size of mCherry-PBP2 (**Supplementary Fig. 8a**). We compared the cell dimensions of the strain to WT, in terms of length, width, and roundness. Cells expressing the mCherry-PBP2 fusion showed a slight increase in width and roundness compared to WT cells, as expected for a slight reduction in PBP2 activity[2] (**Supplementary Fig. 8b-c**). We sequenced the genomic DNA of the strain and found that the *mcherry-pbp2* strain harbored two missense mutations in the *ftsW* gene encoding the glycosyltransferase implicated in division (**Supplementary Fig. 8d)**. To ensure that these mutations in *ftsW* did not affect any observed phenotypes, we conducted backcross experiments to transfer these mutations into the WT background. The resulting strain showed no significant differences in phenotype compared to WT strains in phase contrast microscopy or FDAA dual short-pulse analyses (**Supplementary Fig. 8e-f)**.

Supplementary Tables

Table S1: Strains used in the study

Strain	Description and/or genotype	Reference or source
E. coli		
NEB 5-alpha	*fhuA2Δ(argF-lacZ)U169 phoA glnV44 Φ80Δ(lacZ)M15 gyrA96 recA1 relA1 endA1 thi-1 hsdR17*	NEB
YB 8804	DH5a / pGFPC-5-$zapA_{ex}$	This study
YB 8873	*E. coli* Top10/ pCHYC-4-$zapA_{cc}$	[3]
YB 9852	DH5a / pNPTS139-*mch-Aex-pbp2*	This study
YB 9856	DH5a / pNPTS138-*Aex-ftsW**	This study
C. crescentus		
YB 135	Wild-type strain CB15	[4]
YB 127	Wild-type strain CB15N	[5]
YB 8872	CB15N *zapA::zapA-mcherry*	[6]
YB 9460	CB15N *pbp2::gfp-pbp2*	[7]
CS 606	CB15N Δ*bla*	[8]
A. excentricus		
YB 258	*A. excentricus* AC48	[9]
YB 8871	AC48 z*apA::zapA-gfp*	This study
YB 8806	AC48 *pbp2::mcherry-pbp2*	This study
YB 8807	AC48 *zapA::zapA-gfp pbp2::mcherry-pbp2*	This study
YB 9858	AC48 *ftsW**	This study
Other species		
YB 3934	*Caulobacter henricii*	Brun Lab UdeM
YB 5193	*Brevundimonas diminuta*	Brun Lab UdeM
YB 7710	*Phenylobacterium conjunctum*	Brun Lab UdeM
YB 642	*Asticcacaulis biprosthecum*	[10]
YB 4650	*Rhodobacter capsulatus SB1003*	Bauer Lab[11]

Table S2: Plasmids used in the study

Plasmids		Antibiotic	References
pNPTS139	sacB-containing Litmus 39 derivative suicide vector used for double homologous recombination	Kan	M.R.K. Alley
pNPTS138	sacB-containing Litmus 38 derivative suicide vector used for double homologous recombination	Kan	M.R.K. Alley
pGFPC-5	Integration plasmid used for creating C-terminal fusions to eGFP	Spec/Strep	[12]
pCHYC-5	Integration plasmid used for creating C-terminal fusions to mCherry	Spec/Strep	[12]
pCHYC-4	Integration plasmid used for creating C-terminal fusions to mCherry	Gent	[12]
psfGFPC-5-*zapA*$_{ex}$	pGFPC-5 bearing the C-terminal fragment of *zapA* (*astex_1642*), with the *egfp* gene replaced by *sfgfp*	Spec/Strep	This study
pCHYC-5-*zapA*$_{ex}$	pCHYC-5 bearing the C-terminal fragment of *zapA* (*astex_1642*)	Spec/Strep	This study
pCHYC-4-*zapA*$_{cc}$	pCHYC-4 bearing the C-terminal fragment *zapA* (*ccna_03356*);	Gent	[6]
pNTPS139-*mCherry-PBP2*	pNTPS derivative for in-frame replacement of *pbp2* (*astex_1631*) with *mCherry-pbp2*	Kan	This study
pNTPS138-*Aex-ftsW**	pNTPS derivative for backcrossing *ftsW* mutation	Kan	This study

Table S3. Primers used in the study

Primer ID	Sequence (5′ to 3′)
Aex_pbp2_up_FW	ACTAGTGGGTCGACCGCATGCGCCCTGTTGGCGGTTCTG
Aex_pbp2_up_RV	TGCTCACCATGGAGGGTTCGCTCAATTGAAC
Aex_pbp2-mch_FW	CGAACCCTCCATGGTGAGCAAGGGCGAG
Aex_pbp2-mch_RV	TCCCTCGAGCTTGTACAGCTCGTCCATGC
Aex_pbp2_Nter_FW	GCATGGACGAGCTGTACAAGCTCGAGGGATCCGGATTGAGCGAACCCTCCATC
Aex_pbp2_Nter_RV	TATCGTGGATCCAGAATTCGCTAGCAATTTTCGAGACGTAGCCC
Aex_zapA_FW	GAGACGTCCAATTGCATATGCGTAGCGTCGATCCGCAG
Aex_zapA_RV	TCGAGATCTTAAGGTACCAACCTCGCCAACGAGCTTTTC
Aex-ftsW*up-FW	AATTGAAGCCGGCTGGCGCCAAGCTTAGGAAGCGCGGGTCATTACAGGAAAAGAC
Aex-ftsW*up-RV	AAAAATGACCACCAGCACGAAGCCGAGCGT
Aex-ftsW*down-FW	ACGCTCGGCTTCGTGCTGGTGGTCATTTTT
Aex-ftsW*down-RV	CGTCACGGCCGAAGCTAGCGAATTCTTACCACTGCGTATGATCATCCGG

Table S4: Genome IDs and mode of cell elongation

Strain	Taxonomy ID	Mode of cell elongation
Agrobacterium tumefaciens	176299	Polar[13]
Asticcacaulis aquaticus	2984212	Binary fission[14]
Asticcacaulis benevestitus	1121022	Binary fission[15]
Asticcacaulis biprosthecum	76891	Polar plus unidirectional midcell elongation – This work
Asticcacaulis excentricus	573065	Unidirectional midcell elongation - this work
Brevundimonas diminuta	751586	Bidirectional midcell elongation - this work
Brevundimonas naejangsanensis	588932	Unknown
Brucella abortus	235	Polar[13]
Caulobacter crescentus	565050	Bidirectional midcell elongation – this work
Caulobacter fusiformis	69396	Binary fission[16]
Caulobacter henricii	69395	Bidirectional midcell elongation – this work
Caulobacter segnis	509190	Unknown
Escherichia coli	511145	Dispersed[17]
Henriciella marina	1121949	Binary fission[18]
Hirschia baltica	582402	Budding[19]
Hyphomicrobium denitrificans	582899	Budding[20]
Hyphomonas neptunium	228405	Budding[21-23]
Maricaulis maris	394221	Binary fission[24]
Oceanicaulis alexandrii	1122613	Binary fission[25]
Peiella sedimenti	3061083	Binary fission[26]
Phenylobacterium composti	457173	Unknown
Phenylobacterium conjunctum	1298959	Unidirectional midcell elongation - this work
Phenylobacterium immobile	21	Unknown
Phenylobacterium koreense	266125	Unknown
Phenylobacterium zucineum	450851	Unknown
Rhodobacter capsulatus	1060	Unidirectional midcell elongation - this work
Rhodobacter sphaeroides	557760	Bidirectional midcell elongation[27]
Rhodomicrobium vannielii	648757	Budding[21,22]
Rhodopseudomonas palustris	1076	Budding[21,22]
Robiginitomaculum antarcticum	1123059	Binary fission[28]
Sagittula stellata	388399	Budding ?[13]- No primary reference
Sinorhizobium meliloti	382	Polar[13]

Supplementary References

1 Letunic, I. & Bork, P. Interactive tree of life (iTOL) v3: an online tool for the display and annotation of phylogenetic and other trees. *Nucleic Acids Res* **44**, W242-245, doi:10.1093/nar/gkw290 (2016).

2 Dion, M. F. *et al.* Bacillus subtilis cell diameter is determined by the opposing actions of two distinct cell wall synthetic systems. *Nat Microbiol* **4**, 1294-1305, doi:10.1038/s41564-019-0439-0 (2019).

3 Woldemeskel, S. A., McQuillen, R., Hessel, A. M., Xiao, J. & Goley, E. D. A conserved coiled-coil protein pair focuses the cytokinetic Z-ring in Caulobacter crescentus. *Mol Microbiol* **105**, 721-740, doi:10.1111/mmi.13731 (2017).

4 Poindexter, J. S. Biological properties and classification of the Caulobacter group. *Bacteriological reviews* **28**, 231 (1964).

5 Evinger, M. & Agabian, N. Envelope-associated nucleoid from Caulobacter crescentus stalked and swarmer cells. *Journal of Bacteriology* **132**, 294-301 (1977).

6 Woldemeskel, S. A., McQuillen, R., Hessel, A. M., Xiao, J. & Goley, E. D. A conserved coiled-coil protein pair focuses the cytokinetic Z-ring in Caulobacter crescentus. *Molecular microbiology* **105**, 721-740 (2017).

7 Hocking, J. *et al.* Osmolality-dependent relocation of penicillin-binding protein PBP2 to the division site in Caulobacter crescentus. *Journal of bacteriology* **194**, 3116-3127 (2012).

8 West, L., Yang, D. & Stephens, C. Use of the Caulobacter crescentus genome sequence to develop a method for systematic genetic mapping. *J Bacteriol* **184**, 2155-2166, doi:10.1128/jb.184.8.2155-2166.2002 (2002).

9 Poindexter, J. L. S. & Cohen-Bazire, G. The fine structure of stalked bacteria belonging to the family Caulobacteraceae. *The Journal of cell biology* **23**, 587-607 (1964).

10 Pate, J. L. & Ordal, E. J. The fine structure of two unusual stalked bacteria. *J Cell Biol* **27**, 133-150, doi:10.1083/jcb.27.1.133 (1965).

11 Zappa, S. & Bauer, C. E. The LysR-type transcription factor HbrL is a global regulator of iron homeostasis and porphyrin synthesis in Rhodobacter capsulatus. *Mol Microbiol* **90**, 1277-1292, doi:10.1111/mmi.12431 (2013).

12 Thanbichler, M., Iniesta, A. A. & Shapiro, L. A comprehensive set of plasmids for vanillate- and xylose-inducible gene expression in Caulobacter crescentus. *Nucleic acids research* **35**, e137-e137 (2007).

13 Brown, P. J. *et al.* Polar growth in the Alphaproteobacterial order Rhizobiales. *Proc Natl Acad Sci U S A* **109**, 1697-1701, doi:10.1073/pnas.1114476109 (2012).

14 Lu, H., Chen, L., Kong, L., Huang, L. & Chen, G. Asticcacaulis aquaticus sp. nov., Asticcacaulis currens sp. nov. and Asticcacaulis machinosus sp. nov., isolated from streams in PR China. *Int J Syst Evol Microbiol* **73**, doi:10.1099/ijsem.0.005974 (2023).

15 Vasilyeva, L. V. *et al.* Asticcacaulis benevestitus sp. nov., a psychrotolerant, dimorphic, prosthecate bacterium from tundra wetland soil. *Int J Syst Evol Microbiol* **56**, 2083-2088, doi:10.1099/ijs.0.64122-0 (2006).

16 Staley, J. T., Bont, J. A. & Jonge, K. Prosthecobacter fusiformis nov. gen. et sp., the fusiform caulobacter. *Antonie Van Leeuwenhoek* **42**, 333-342, doi:10.1007/bf00394132 (1976).

17 Kuru, E. *et al.* In Situ probing of newly synthesized peptidoglycan in live bacteria with fluorescent D-amino acids. *Angew Chem Int Ed Engl* **51**, 12519-12523, doi:10.1002/anie.201206749 (2012).

18 Quan, Z. X. *et al.* Henriciella marina gen. nov., sp. nov., a novel member of the family Hyphomonadaceae isolated from the East Sea. *J Microbiol* **47**, 156-161, doi:10.1007/s12275-008-0290-0 (2009).

19 Schlesner, H., Bartels, C., Sittig, M., Dorsch, M. & Stackebrandt, E. Taxonomic and phylogenetic studies on a new taxon of budding, hyphal Proteobacteria, Hirschia baltica gen. nov., sp. nov. *Int J Syst Bacteriol* **40**, 443-451, doi:10.1099/00207713-40-4-443 (1990).

20 URAKAMI, T., SASAKI, J., SUZUKI, K.-I. & KOMAGATA, K. Characterization and Description of Hyphomicrobium denitrificans sp. nov. *International Journal of Systematic and Evolutionary Microbiology* **45**, 528-532, doi:https://doi.org/10.1099/00207713-45-3-528 (1995).

21 Moore, R. L. The biology of Hyphomicrobium and other prosthecate, budding bacteria. *Annu Rev Microbiol* **35**, 567-594, doi:10.1146/annurev.mi.35.100181.003031 (1981).

22 Hirsch, P. Budding bacteria. *Annu Rev Microbiol* **28**, 391-444, doi:10.1146/annurev.mi.28.100174.002135 (1974).

23 Cserti, E. *et al.* Dynamics of the peptidoglycan biosynthetic machinery in the stalked budding bacterium Hyphomonas neptunium. *Mol Microbiol* **103**, 875-895, doi:10.1111/mmi.13593 (2017).

24 Abraham, W. R. *et al.* Phylogeny and polyphasic taxonomy of Caulobacter species. Proposal of Maricaulis gen. nov. with Maricaulis maris (Poindexter) comb. nov. as the type species, and emended description of the genera Brevundimonas and Caulobacter. *Int J Syst Bacteriol* **49 Pt 3**, 1053-1073, doi:10.1099/00207713-49-3-1053 (1999).

25 Strömpl, C. *et al.* Oceanicaulis alexandrii gen. nov., sp. nov., a novel stalked bacterium isolated from a culture of the dinoflagellate Alexandrium tamarense (Lebour) Balech. *Int J Syst Evol Microbiol* **53**, 1901-1906, doi:10.1099/ijs.0.02635-0 (2003).

26 Wu, N. *et al.* Peiella sedimenti gen. nov., sp. nov., a novel taxon within the family Caulobacteraceae isolated from sediment of a river. *International Journal of Systematic and Evolutionary Microbiology* **74**, doi:https://doi.org/10.1099/ijsem.0.006344 (2024).

27 Lakey, B. D. *et al.* The role of CenKR in the coordination of <i>Rhodobacter sphaeroides</i> cell elongation and division. *mBio* **14**, e00631-00623, doi:doi:10.1128/mbio.00631-23 (2023).

28 Lee, K., Lee, H. K., Choi, T. H. & Cho, J. C. Robiginitomaculum antarcticum gen. nov., sp. nov., a member of the family Hyphomonadaceae, from Antarctic seawater. *Int J Syst Evol Microbiol* **57**, 2595-2599, doi:10.1099/ijs.0.65274-0 (2007).
