# Supplementary material for: Phenotypic plasticity in cell elongation among closely related bacterial species
Source: Nat Commun. 2025 Jun 2;16:5099. doi: 10.1038/s41467-025-60005-y (PMC12130487; doi:10.1038/s41467-025-60005-y)
Supplement: Supplementary file 2 — Reporting Summary [file 41467_2025_60005_MOESM2_ESM.pdf]

nature portfolio

Corresponding author(s): Yves Brun

Last updated by author(s): Apr 15, 2025

Reporting Summary

Nature Portfolio wishes to improve the reproducibility of the work that we publish. This form provides structure for consistency and transparency in reporting. For further information on Nature Portfolio policies, see our Editorial Policies and the Editorial Policy Checklist.

Statistics

For all statistical analyses, confirm that the following items are present in the figure legend, table legend, main text, or Methods section.

n/a	Confirmed	
☐	☒	The exact sample size (*n*) for each experimental group/condition, given as a discrete number and unit of measurement
☐	☒	A statement on whether measurements were taken from distinct samples or whether the same sample was measured repeatedly
☐	☒	The statistical test(s) used AND whether they are one- or two-sided<br>*Only common tests should be described solely by name; describe more complex techniques in the Methods section.*
☒	☐	A description of all covariates tested
☒	☐	A description of any assumptions or corrections, such as tests of normality and adjustment for multiple comparisons
☐	☒	A full description of the statistical parameters including central tendency (e.g. means) or other basic estimates (e.g. regression coefficient) AND variation (e.g. standard deviation) or associated estimates of uncertainty (e.g. confidence intervals)
☐	☒	For null hypothesis testing, the test statistic (e.g. *F*, *t*, *r*) with confidence intervals, effect sizes, degrees of freedom and *P* value noted<br>*Give P values as exact values whenever suitable.*
☐	☒	For Bayesian analysis, information on the choice of priors and Markov chain Monte Carlo settings
☒	☐	For hierarchical and complex designs, identification of the appropriate level for tests and full reporting of outcomes
☒	☐	Estimates of effect sizes (e.g. Cohen's *d*, Pearson's *r*), indicating how they were calculated

Our web collection on statistics for biologists contains articles on many of the points above.

Software and code

Policy information about availability of computer code

Data collection	Microscopy images were acquired using Nikon NIS Elements software. Protein band detection and gel imaging were performed using Image Lab (version 6.0.1 build 34, Bio-Rad Laboratories). Sequencing data were collected using MinKNOW (v21.05.12) for Oxford Nanopore sequencing and bcl-convert (v3.9.30) for Illumina sequencing. For genome and protein sequence retrieval and annotation, publicly available resources such as BLAST (https://blast.ncbi.nlm.nih.gov/Blast.cgi) and UniProt (https://www.uniprot.org/) were used.
Data analysis	Image analysis was performed using FIJI (version 2.14.0/1.54f) and the MicrobeJ plugin (v5.13) to obtain cell dimensions, fluorescence intensities, kymographs, and demographs. GraphPad Prism (v10.3.0) was used to generate histograms, fluorescence intensity profiles, and perform statistical analyses. Phylogenetic analyses were carried out using PhyloSift, MrBayes, RAxML (v8.2.10), MUSCLE (v3.8.31), and PhyML (v3.2). Genome annotation was conducted using Prokka, and variant calling was performed using Breseq (v0.37.1). Nanopore sequencing data were processed using Guppy (v6.1.1), Trycycler (v0.5.3), Medaka (v1.6.0), Canu (v2.2), Flye (v2.9.1), and Miniasm (v0.3). Assembly quality was assessed using Quast (v5.2.0) and BUSCO (v5.4.0). Synteny and operon organization analyses were performed using BEDTools, MCL, and visualized with EasyFig. All graphical output and figure assembly were finalized using Adobe Illustrator CC 2023.

For manuscripts utilizing custom algorithms or software that are central to the research but not yet described in published literature, software must be made available to editors and reviewers. We strongly encourage code deposition in a community repository (e.g. GitHub). See the Nature Portfolio guidelines for submitting code & software for further information.

Data

Policy information about availability of data

All manuscripts must include a data availability statement. This statement should provide the following information, where applicable:

- Accession codes, unique identifiers, or web links for publicly available datasets
- A description of any restrictions on data availability
- For clinical datasets or third party data, please ensure that the statement adheres to our policy

Data generated in the study are in the source data file. Accession codes for whole genome sequencing data of A. biprosthecum and P. conjunctum are in the Data Availability section. The raw sequencing reads generated in this study have been deposited in the NCBI Sequence Read Archive (SRA) under accession code PRJNA1231080. The whole genome sequencing and variant calling analysis of A. excentricus mch-pbp2 generated in this study have been deposited in Figshare at https://doi.org/10.6084/m9.figshare.27623046. Source data are provided with this paper.

Research involving human participants, their data, or biological material

Policy information about studies with human participants or human data. See also policy information about sex, gender (identity/presentation), and sexual orientation and race, ethnicity and racism.

Reporting on sex and gender	*Use the terms sex (biological attribute) and gender (shaped by social and cultural circumstances) carefully in order to avoid confusing both terms. Indicate if findings apply to only one sex or gender; describe whether sex and gender were considered in study design; whether sex and/or gender was determined based on self-reporting or assigned and methods used. Provide in the source data disaggregated sex and gender data, where this information has been collected, and if consent has been obtained for sharing of individual-level data; provide overall numbers in this Reporting Summary. Please state if this information has not been collected. Report sex- and gender-based analyses where performed, justify reasons for lack of sex- and gender-based analysis.*
Reporting on race, ethnicity, or other socially relevant groupings	*Please specify the socially constructed or socially relevant categorization variable(s) used in your manuscript and explain why they were used. Please note that such variables should not be used as proxies for other socially constructed/relevant variables (for example, race or ethnicity should not be used as a proxy for socioeconomic status). Provide clear definitions of the relevant terms used, how they were provided (by the participants/respondents, the researchers, or third parties), and the method(s) used to classify people into the different categories (e.g. self-report, census or administrative data, social media data, etc.) Please provide details about how you controlled for confounding variables in your analyses.*
Population characteristics	*Describe the covariate-relevant population characteristics of the human research participants (e.g. age, genotypic information, past and current diagnosis and treatment categories). If you filled out the behavioural & social sciences study design questions and have nothing to add here, write "See above."*
Recruitment	*Describe how participants were recruited. Outline any potential self-selection bias or other biases that may be present and how these are likely to impact results.*
Ethics oversight	*Identify the organization(s) that approved the study protocol.*

Note that full information on the approval of the study protocol must also be provided in the manuscript.

Field-specific reporting

Please select the one below that is the best fit for your research. If you are not sure, read the appropriate sections before making your selection.

☒ Life sciences ☐ Behavioural & social sciences ☐ Ecological, evolutionary & environmental sciences

For a reference copy of the document with all sections, see nature.com/documents/nr-reporting-summary-flat.pdf

Life sciences study design

All studies must disclose on these points even when the disclosure is negative.

Sample size	No sample size calculation or statistical method was used to predetermine sample sizes. Sample sizes ranged from 160 to 2208 cells, depending on the experiment, and are specified in the corresponding figure panels. These sample sizes were chosen based on standard practices in the field and were sufficient to observe reproducible trends across independent replicates. For short pulse analyses, 50 cells were selected to plot the fluorescence intensities of the two FDAAs along the cell length. For pulse-chase experiments involving the ZapA fluorescent signal, 25 cells were selected to visualize and quantify FDAA fluorescence profiles along the cell length.
Data exclusions	The images and the fields of cells analyzed were selected randomly. Cells out of focus or inaccurately segmented were excluded from the analysis. Cells without holdfast staining or in the process of division were excluded from the analysis. All filtering processes were aplied uniformly. Cells were excluded from the analysis when cells did not show fluorescence signal for mCh-Pbp2.

Replication	All microscopy experiments were repeated at least three times. Analyses either represents the pooled data or a representative experiment as described in figure legends or methods.
Randomization	In figure 4, ShapePlots are presented individually for four categories of cells binned by cell length. For other experiments, after data collection, all measurments and analyses were performed identically.
Blinding	Data analysis was not performed blindly; however, image processing and analysis were carried out using MicrobeJ with automated, unbiased procedures, and identical settings were applied wherever possible.

Reporting for specific materials, systems and methods

We require information from authors about some types of materials, experimental systems and methods used in many studies. Here, indicate whether each material, system or method listed is relevant to your study. If you are not sure if a list item applies to your research, read the appropriate section before selecting a response.

Materials & experimental systems

n/a	Involved in the study
☐	☒ Antibodies
☒	☐ Eukaryotic cell lines
☒	☐ Palaeontology and archaeology
☒	☐ Animals and other organisms
☒	☐ Clinical data
☒	☐ Dual use research of concern
☒	☐ Plants

Methods

n/a	Involved in the study
☒	☐ ChIP-seq
☒	☐ Flow cytometry
☒	☐ MRI-based neuroimaging

Antibodies

Antibodies used	Anti-RFP antiserum (Chen et al., 2005) was used at dilution of 1:2,500.
Validation	The specificity of the antibody used was verified by immunoblot analysis of reference strains expressing the indicated protein, which is absent in the WT strain.

Plants

Seed stocks	N/A
Novel plant genotypes	N/A
Authentication	N/A
